# Abscisic Acid Is a Major Regulator of Grape Berry Ripening Onset: New Insights into ABA Signaling Network

**DOI:** 10.3389/fpls.2017.01093

**Published:** 2017-06-21

**Authors:** Stefania Pilati, Giorgia Bagagli, Paolo Sonego, Marco Moretto, Daniele Brazzale, Giulia Castorina, Laura Simoni, Chiara Tonelli, Graziano Guella, Kristof Engelen, Massimo Galbiati, Claudio Moser

**Affiliations:** ^1^Research and Innovation Centre, Fondazione Edmund MachSan Michele all′Adige, Italy; ^2^Dipartimento di Bioscienze, Università degli Studi di MilanoMilan, Italy; ^3^Department of Physics, Bioorganic Chemistry Lab, University of TrentoTrento, Italy; ^4^Istituto di Biofisica, Consiglio Nazionale delle RicercheTrento, Italy

**Keywords:** Abscisic acid (ABA), grapevine (*Vitis vinifera*), berry ripening, RNA sequencing, promoter analysis, AREB/ABF

## Abstract

Grapevine is a world-wide cultivated economically relevant crop. The process of berry ripening is non-climacteric and does not rely on the sole ethylene signal. Abscisic acid (ABA) is recognized as an important hormone of ripening inception and color development in ripening berries. In order to elucidate the effect of this signal at the molecular level, pre-véraison berries were treated *ex vivo* for 20 h with 0.2 mM ABA and berry skin transcriptional modulation was studied by RNA-seq after the treatment and 24 h later, in the absence of exogenous ABA. This study highlighted that a small amount of ABA triggered its own biosynthesis and had a transcriptome-wide effect (1893 modulated genes) characterized by the amplification of the transcriptional response over time. By comparing this dataset with the many studies on ripening collected within the grapevine transcriptomic compendium Vespucci, an extended overlap between ABA- and ripening modulated gene sets was observed (71% of the genes), underpinning the role of this hormone in the regulation of berry ripening. The signaling network of ABA, encompassing ABA metabolism, transport and signaling cascade, has been analyzed in detail and expanded based on knowledge from other species in order to provide an integrated molecular description of this pathway at berry ripening onset. Expression data analysis was combined with *in silico* promoter analysis to identify candidate target genes of ABA responsive element binding protein 2 (VvABF2), a key upstream transcription factor of the ABA signaling cascade which is up-regulated at véraison and also by ABA treatments. Two transcription factors, VvMYB143 and VvNAC17, and two genes involved in protein degradation, Armadillo-like and Xerico-like genes, were selected for *in vivo* validation by VvABF2-mediated promoter *trans*-activation in tobacco. VvNAC17 and Armadillo-like promoters were induced by ABA via VvABF2, while VvMYB143 responded to ABA in a VvABF2-independent manner. This knowledge of the ABA cascade in berry skin contributes not only to the understanding of berry ripening regulation but might be useful to other areas of viticultural interest, such as bud dormancy regulation and drought stress tolerance.

## Introduction

Grape is a traditional world-wide cultivated crop, whose fruit is consumed fresh or dried as table grapes, fermented to produce wines, spirits and vinegar or transformed into pharmaceutical health-promoting products. The process of fruit development has been intensively studied initially to improve quality of production and more recently to maintain high quality under changing climatic conditions. Grape berry development can be divided into two phases of berry growth: an initial phase from fruit set until green hard berries, characterized by embryo maturation in the seeds, pericarp intense cell duplication, malic and tartaric acid accumulation and proanthocyanidin synthesis, and a final phase of ripening, characterized by fruit softening, mesocarp cell enlargement and sugar and aroma accumulation and skin coloring. The onset of ripening, that is the transition from the first to the second phase, implies an extensive reprogramming of the berry transcriptome, as observed in several “-omic” studies (Deluc et al., 2007; Pilati et al., 2007; Fasoli et al., 2012; Lijavetzky et al., 2012). An integrated network analysis recently highlighted that this transition occurs via an extensive gene down-regulation driving the suppression of the vegetative growth metabolism and the activation of maturation-specific pathways (Palumbo et al., 2014).

Such transcriptional and metabolic reprogramming is orchestrated by numerous signals, such as hormones, in particular ABA, ethylene and brassinosteroids, reviewed in (Kuhn et al., 2014), physiological modifications, such as cell turgor and elasticity (Castellarin et al., 2011), and metabolic factors, such as sugar and reactive oxygen species accumulation (Gambetta et al., 2010; Pilati et al., 2014). However, their reciprocal influence on ripening inception has not been disentangled so far, due to the complexity of the system. Recently, Castellarin et al. (2015) proposed a timeline of events leading to the onset of ripening: an initial fall of elasticity and turgor pressure in the berry is followed by ABA and sugar accumulation and then skin coloring. Yet, an ABA sharp increase at ripening onset has been reported in numerous studies (Deluc et al., 2007; Wheeler et al., 2009; Gambetta et al., 2010; Sun et al., 2010). The fast accumulation of ABA in the cells is due the its typical positive feedback loop, triggered by a small amount of hormone coming possibly from the leaves (Antolìn et al., 2003) or by diffusion from the mature, dormancy-acquiring seeds (Kondo and Kawai, 1998). This ripening-specific accumulation is reported also in peach, sweet cherry and tomato (Zhang et al., 2009; Sun et al., 2012; Tijero et al., 2016).

Abscisic acid regulates a variety of plant developmental processes, such as leaf senescence, seed maturation and dormancy, bud dormancy and adaptive responses to abiotic and biotic stresses, in particular drought and salinity, by means of stomata closure, osmotic potential regulation and/or wax deposition (Nambara and Kuchitsu, 2011). During plant evolution, ABA conserved its ancestral role in cellular responses modulation to stimuli affecting the cell water status, but acquired new functions in the regulation of different processes, sometimes also in a species specific way (Takezawa et al., 2011; Wanke, 2011). In fleshy fruit ripening, the relationship between ABA and sugar accumulation and turgor pressure, which together determine water uptake and cell enlargement, could represent an example of acquired functionality of ABA in angiosperms (Wheeler et al., 2009; Gambetta et al., 2010). In tomato, both a transgenic line blocked in ABA synthesis and one overexpressing a transcription factor activating ABA response demonstrated the effect of ABA on tomato texture and firmness and primary metabolites accumulation (Sun et al., 2012; Bastías et al., 2014). A similar decrease in fruit firmness was observed transforming tomato with the Vitis homolog of this transcription factor (Nicolas et al., 2014). ABA effect on berry skin coloring was highlighted in studies focused on seedless varieties of table grapes, where the absence of seeds correlated with low amounts of ABA and low color development, recovered by ABA treatments (Kondo and Kawai, 1998; Ferrara et al., 2013). Nonetheless, studies focused on the effects of adverse external conditions highlighted that anthocyanin accumulation in condition of water stress required not only ABA, but also sugar accumulation and possibly other stimuli, suggesting that ABA is not the only necessary signal for color development, or it can exert an indirect effect (Castellarin et al., 2007; Wheeler et al., 2009; Gambetta et al., 2010; Ferrandino and Lovisolo, 2014).

Abscisic acid signaling network encompasses genes involved in its biosynthesis, degradation, conjugation and transport, whose reciprocal transcription and enzymatic activities determine ABA cellular content, and genes involved in its perception and signaling cascade. The knowledge of this network has burst recently, taking advantage of the combination of molecular, biochemical, forward and reverse genetic studies in Arabidopsis, reviewed in (Finkelstein, 2013) and the availability of the genome sequence of *Vitis vinifera* (Jaillon et al., 2007; Velasco et al., 2007), which accelerated the transfer of knowledge from the model plant to this crop. The early steps of ABA biosynthesis take place in the plastid as part of the MEP pathway leading to carotenoids production. NCED catalyzes the first committed step in ABA biosynthesis, and is rate-limiting, reviewed in (Nambara and Marion-Poll, 2005). NCEDs are encoded by multigene families and the expression of the specific family members is tightly regulated in response to stress or developmental signals contributing to ABA synthesis in different contexts. In addition to ABA synthesis, catabolism is a major mechanism for regulating ABA levels: ABA can be irreversibly hydroxylated at the 8′ position by P-450 type monooxygenases to give an unstable intermediate (8′-OH-ABA) isomerized to phaseic acid; or reversibly esterified to ABA-glucose ester (ABA-GE), which can accumulate in vacuoles or apoplast as storage. Transporters of the G subfamily of the ATP-binding cassette (ABC) family mediate the import and export of ABA through the plasmalemma (Jarzyniak and Jasinski, 2014).

Abscisic acid perception and signaling in grapevine has been recently elucidated in root and leaf (Boneh et al., 2011, 2012) and in fruit (Gambetta et al., 2010), by identifying and partially characterizing ABA receptors, PP2Cs and SnRK2 kinases. The best characterized ABA receptors in Arabidopsis are soluble proteins of the family PYR (pyrabactin resistant), PYL (PYR-like) or RCAR (regulatory component of ABA receptor). Eight RCARs, four of which were induced by ABA in leaf, were identified in grapevine (Boneh et al., 2011). ABA binds to PYR/PYL/RCAR proteins, resulting in a conformational change that enhances stability of a complex with clade A PP2Cs, which in Arabidopsis are all induced by ABA and include the ABA insensitive mutants *abi1* and *abi2* (Merlot et al., 2001). In grapevine, nine PP2Cs have been identified (Gambetta et al., 2010; Boneh et al., 2011): six are induced by ABA in leaf, and five are induced at véraison in the berry. In the absence of ABA, the PP2Cs keep SNF1-related kinases (SnRKs) inactive through physical interaction and dephosphorylation. When ABA binds to its receptors, they recruit PP2C, thus releasing the inhibition of SnRKs which become active by autophosphorylation and activate more than 50 target proteins, which include transcription factors as well as other targets. In grapevine, seven SnRKs were identified and they appeared differently modulated in leaf, root and fruit upon abiotic stresses and development. PP2Cs may also dephosphorylate other classes of kinases, e.g., the ABA-stimulated calcium-dependent protein kinase (ACDK), linking ABA to calcium signaling (Yu et al., 2006) and widening the cascade. Among the transcription factors activated by ABA, a subgroup of the bZIP family, called AREB/ABF (Liu et al., 2014), are directly activated by SnRKs (Yoshida et al., 2010): in grapevine 11 putative ABA-responsive bZIPs have been identified by sequence homology and one of them, VvABF2, has been recently characterized (Nicolas et al., 2014).

The present work analyzes the early transcriptional events occurring in green hard, pre-véraison berry skin treated with exogenous ABA showing the dramatic effect of this hormone on ripening onset and identifying candidates targets of VvABF2, thus expanding our knowledge on ABA network in the fruit.

## Materials and Methods

### Plant Material, Biochemical Analyses, ABA Treatment

During 2011, two clusters of *V. vinifera* cv. Pinot Noir ENTAV115 were collected almost daily between 9 am and 10 am at 6–7 weeks post-flowering (wpf), corresponding to EL-33 and EL-34, at the FEM study site (San Michele all′Adige-TN Italy). Each cluster represented one biological replicate. Each cluster was divided in smaller bunches and then half of them, at random, were pressed for must analysis by means of Fourier transform infrared spectroscopy (FTIR) using the instrument WineScanTM Type 77310 (Foss Electric, Denmark) while the remaining small bunches were rapidly frozen. Frozen berries were peeled with a scalpel and the skins were ground to obtain a fine powder. Skin anthocyanin concentration was measured after methanol extraction (1 g berry skin powder in 10 mL methanol) according to the double pH differential method (Cheng and Breen, 1991). Lipid extraction and analysis were performed as described in Pilati et al. (2014). ABA detection and quantification was carried by LC-UV-MS technique. In particular we used as stationary phase a column Kinetex C18 (5 μm, 150 mm × 4.6 mm, flow 0.8 mL/min) and as mobile phase an A:B gradient elution (A = H_2_O + 0.5% formic acid; B = MeOH + 0.5% formic Acid) with B changing from 40 to 55% in 10 min. 10 μL of pure ABA solution or raw skin berry extracts (solution prepared in MeOH/CHCl_3_ 9:1) were injected in every chromatographic run. The retention time of ABA was 8.9 min. ABA was detected both by ion-positive mode ESI-MS analysis (characteristic ions at m/z 287, 265, 247, 229, 201, 187, and 173) and by photodiode array detector. ABA was quantified by interpolation on a working curve (absorbance vs. concentration) built on three ABA solutions at concentration 1.9, 19, and 190 ng/μL and the corresponding absorbance measured at fixed wavelength of 262 nm (*R*^2^ = 0.998). During 2013, three clusters of *V. vinifera* cv. Pinot Noir ENTAV115 were collected between 9 and 10 am at 7 wpf, corresponding to EL-33, at the FEM study site (San Michele all′Adige-TN Italy). Each cluster represented one biological replicate. Berries were detached from each cluster by cutting the petiole, at 2–3 mm distance from the berry. Berries were washed in 0.1% Plant Preservative Mixture (PPM, Duchefa) water solution for 30 min. 20 berries per cluster were put in a 100 mL water solution containing 0.2 mM ABA (Sigma) and 0.5% methanol (used to dissolve ABA) or 0.5% methanol as control. After 20 h mild shaking, the berries were extensively rinsed and then eight berries were frozen in liquid nitrogen. The remaining berries, both treated and not treated with ABA, were plated on solid medium in Petri dishes (0.9% agar, 10% sucrose, 0.1% PPM) for 24 h, and then eight were frozen as described above. ABA and sucrose concentrations were taken from Gambetta et al. (2010).

### RNA Extraction and Expression Analysis by qPCR

Total RNA was extracted from the skin powder samples using Spectrum Total Plant RNA kit (Sigma) and was quantified using a Nanodrop 8000 (Thermo Scientific). The integrity was checked using Bioanalyzer 2100 (Agilent) and RNA Nano Chips. For Real-time PCR analyses, first strand cDNA was synthesized from 2 μg RNA using the SuperScript VILO cDNA Synthesis Kit (Invitrogen) according to the manufacturer’s instructions. The cDNAs were mixed with Fast SYBR Green Master Mix (Applied Biosystems) and amplified on a ViiA 7 Real Time PCR System (Applied Biosystems) using an initial heating step at 95°C for 20 min, followed by 40 cycles of 95°C for 1 min and 60°C for 20 s, using the primers reported in Supplementary Table S1. Raw fluorescence data were extracted using Viia 7 Software v1.0. Ct and reaction efficiency were calculated using LinRegPCR (Ruijter et al., 2009). Relative expression was calculated according to (Pfaffl, 2001) by centering expression values for each gene on the mean value. Three reference genes (Actin, SAND and GAPDH) were used for normalization with geNorm (Vandesompele et al., 2002). For RNA sequencing, 2 μg RNA for each sample were shipped in dry ice to Genomicx4life (Salerno, Italy).

### RNA-Seq Analysis and Identification of Differentially Expressed Genes

Sequencing has been performed on Illumina Hi-seq 1500, producing 100 nt directional single-end reads. Raw reads were pre-processed for quality using fastqc v.0.11.2^1^ and cleaned with cutadapt v.1.12 (Martin, 2011). The resulting reads were aligned to the grape (12x v1^2^) genome using the Subread aligner (Liao et al., 2013). Raw read counts were extracted from the Subread alignments using the featureCount read summarization program (Liao et al., 2014). The summarized read count data was used to identify DEGs among various treatments by using the voom method (Law et al., 2014), which estimates the mean-variance relationship of the log-counts, generating a precision weight for each observation that is fed into the limma empirical Bayes analysis pipeline (Smyth, 2004). A Volcano Plot generated using the ShinyVolcanoPlot Web App^3^ was used to select sets of DEGs for each comparison based on both *p*-value and expression fold change. In the present work, a maximum *p*-value of 0.05 and a minimum absolute fold change of 2 were imposed. Raw sequences were deposited at the Sequence Read Archive of the National Center for Biotechnology^4^ under BioProject accession number PRJNA369777.

### Gene Annotation and Promoter Analysis

Vitisnet gene annotation has been used (Grimplet et al., 2009), except for the transcription factors gene families already characterized in grapevine, for which the specific published annotation has been used (Licausi et al., 2010; Wang N. et al., 2013; Liu et al., 2014; Wang et al., 2014; Wong et al., 2016). Functional class enrichment was performed on GO (Gene Ontology) terms (annotation^5^) using the TopGO Bioconductor package (Alexa and Rahnenfuhrer, 2016) and on Vitisnet gene annotation (Grimplet et al., 2009) taking advantage of the VESPUCCI grape compendium (Moretto et al., 2016). Promoter analysis has been performed on the 1-kb promoters of the genes modulated at 20 and 44 h using DREME software (Bailey, 2011). Statistically enriched motifs were annotated using the “DAP motifs” database for Arabidopsis (O’Malley et al., 2016). The enriched motifs were searched and counted in the dataset using Patmatch software (Yan et al., 2005).

### Plasmid Constructs

Two type of constructs were prepared for transient expression, using the Gateway system (Karimi et al., 2002). The coding sequence of ABF2 (VIT_18s0001g10450) was amplified from Pinot Noir berry cDNA using Phusion DNA polymerase (Finnzymes) and the primers ABF2Fw and ABF2rev and cloned into pENTR-D-TOPO vector (Invitrogen), sequenced and transferred into pK7WG2, under the control of 35S promoter. The 1-kb promoters of VvNAC17 (VIT_19s0014g03290), Armadillo-like (VIT_17s0000g08080), Xerico-like (VIT_01s0137g00780), VvMYB143 (VIT_00s0203g00070) and HB5 (VIT_04s0023g01330) were amplified from PN40024 genomic DNA using Phusion DNA polymerase and the pairs of primers indicated in Supplementary Table S1. These DNA fragments were cloned in pENTR-D-TOPO, sequenced and transferred into PHGWFS7, upstream of EGFP and GUS reporter genes.

### Transient Expression Assay in *N. benthamiana*

Promoter activation assays were performed in 5-week-old *Nicotiana benthamiana* plants agroinfiltrated as described in Li (2011). Three leaves from four tobacco plants, representing four biological replicates, were co-infiltrated with the activating plasmid pK7WG2:CaMV35S:ABF2 and individual pHGWFS7:promoter:GUS target constructs. Leaves co-infiltrated with the pK7WG2 empty vector and each of the pHGWFS7:promoter:GUS plasmids were used as a control for the *trans*-activation assay. 48 h after the first infiltration, leaves were infiltrated with 50 μM ABA dissolved in 10 mM MgCl_2_ and 0.07% EtOH, or with a mock solution (0.07% EtOH in 10 mM MgCl_2_). Leaf samples were collected at 15 min, 1 and 3 h after the beginning of the ABA treatment. qPCR analysis of GUS expression was performed as previously described for the grape samples, using the primers qPCR_GUSF1 and qPCR_GUSR1 (Supplementary Table S1). GUS expression was normalized using the *Elongation factor 1*α (*EF-1*α) gene (AF120093), amplified with primers pEFfw and pEFrev (Schmidt and Delaney, 2010).

## Results

### Treatment of Pre-véraison Berries with ABA

**FIGURE 1:**
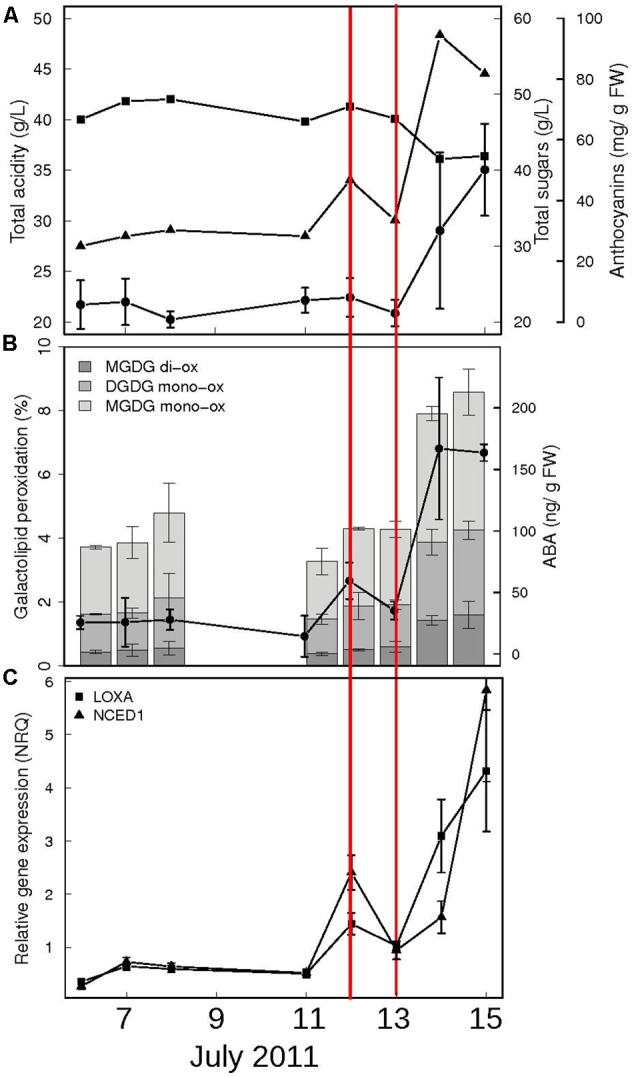
Biochemical and molecular profiling of Pinot Noir berries at the onset of ripening (season 2011). **(A)** Mean values of total acids (squares, expressed as grams of tartaric acid per liter) and sugars (triangles, expressed as total soluble solids in °Brix) of the must obtained from two clusters, at each time point. Berry skins anthocyanin content (circles) is expressed as grams of mg cyanidin-3-glucoside per gram of berry fresh weight. **(B)** Total galactolipid peroxidation (stacked bars) and ABA content (circles) in berry skins. The mono-oxidized and di-oxidized forms of MGDG 36:6 and DGDG 36:6 are shown as percentage of total MGDG and DGDG, respectively. Data are means of two biological replicates ± SD. **(C)** PnLOXA (squares) and NCED1 (triangles) gene expression in berry skins. Normalized relative quantities ± SE were calculated using three reference genes; *n* = 2. The *x*-axis represents actual sampling date during July 2011. The precise moment preceding ripening start is indicated between red lines (July, 12th).

**FIGURE 2:**
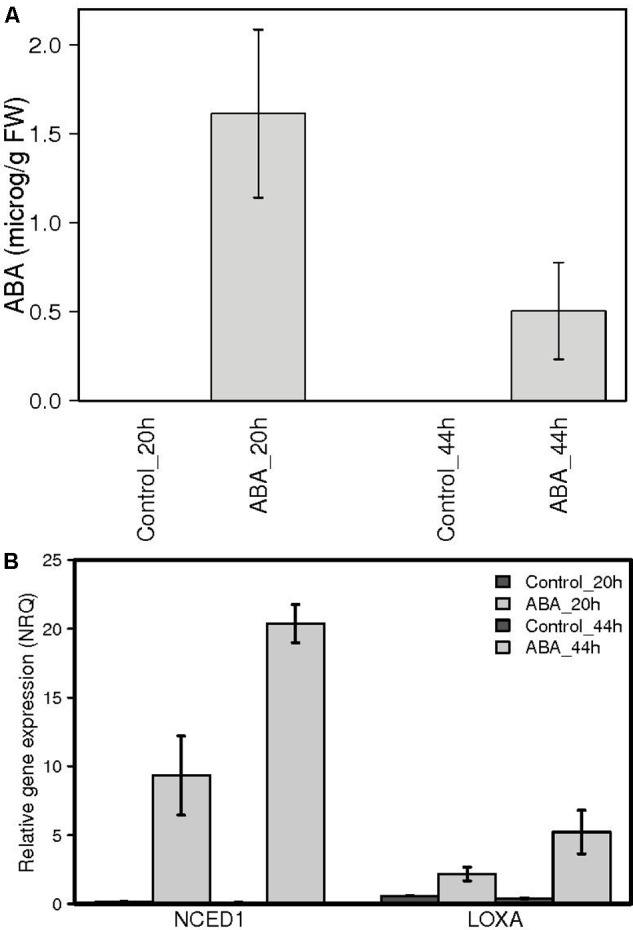
Abscisic acid quantification and LOXA and NCED1 gene expression analysis in ABA treated berry skins. **(A)** ABA was measured by HPLC-MS. No ABA was detected in the untreated samples. Means of two biological replicates are represented. **(B)** PnLOXA and NCED1 gene expression in the skin of berries treated with 0.2 mM ABA for 20 h and then cultured on solid 10% sucrose medium without ABA for 24 h (light gray) and controls (dark gray). Normalized relative quantities were obtained by RT-PCR analysis using the two best reference genes. Data represent the mean of three biological replicates.

In order to identify the exact moment preceding the onset of ripening, we focused our attention on the 2 weeks preceding color break in Pinot Noir berries. Samples were collected daily during season 2011 and analyzed for biochemical and molecular parameters which are known from both literature and our experience to change dramatically at ripening inception (Pilati et al., 2014). These include biochemical profiles, such as total acidity, sugar and anthocyanins content (**Figure 1A**), galactolipid peroxidation state and ABA content in berry skins (**Figure 1B**) and gene expression profiles, such as those of lipoxygenase A (LOXA, VIT_06g0004s01510) – responsible of the enzymatic galactolipid peroxidation – and of NCED1 (VIT_19s0093g00550) – first committed enzyme in ABA biosynthesis (**Figure 1C**). This preliminary analysis showed that in the 48 h preceding anthocyanins accumulation (on July 14th), all these parameters undergo a transition, which marks the beginning of a distinct developmental phase, i.e., the ripening. This discontinuity implies an extensive regulation occurring within the cells to trigger all the metabolic pathways characterizing the biochemical changes of fruit ripening. To study the role of ABA as a trigger of ripening, in 2013 we collected berries at the hard green pre-véraison stage (E-L 33) and treated them with exogenous ABA. The treatment was performed on detached berries with short petioles in an aqueous medium containing 0.2 mM ABA for 20 h, which allowed for both homogenous ABA diffusion into the berry skin trough functional stomata and accurate experimental reproducibility. After 20 h, eight berries were collected, rinsed with water and frozen while the remaining were rinsed and plated for additional 24 h on solid medium containing 10% sucrose, in the absence of ABA, and then frozen (**Supplementary Figure S1**). Sucrose was added for avoiding osmotic stress according to (Gambetta et al., 2010) in order to mimic berry sugar content at véraison (6–7°Brix). Treatment efficacy has been verified by two independent approaches. Firstly, ABA was quantified in skin samples of control and ABA treated berries by HPLC-UV-MS showing an average accumulation of 1.62 micrograms ABA/gr FW skin powder at 20 h and 0.55 μg/gr FW at 44 h in treated samples (**Figure 2A**). The latter value was similar to physiological values measured at véraison in other studies, such as 300 ng/gr FW in Wheeler et al. (2009) and 200 ng/gr FW in Sun et al. (2010), whereas higher ABA level at 20 h could be explained by ABA direct uptake. The very low ABA measured in control samples is consistent with berries at the green hard pre-véraison stage (**Figure 1B**). Secondly, the expression of the two genes known to be up-regulated at véraison, LOXA and NCED1, has been measured by qPCR (**Figure 2B**). NCED1 was up-regulated 66 times by ABA treatment at 20 h and 198 times at 44 h; LOXA showed a similar behavior, as it was up-regulated 4 and 13 times at 20 and 44 h, respectively. These profiles are consistent with those observed in 2011 in the transition from E-L 33 to E-L 34.

Moreover, the induction of NCED1 can explain the intracellular ABA level measured at 44 h.

### ABA Extensively Modulates the Berry Skin Transcriptome

**FIGURE 3:**
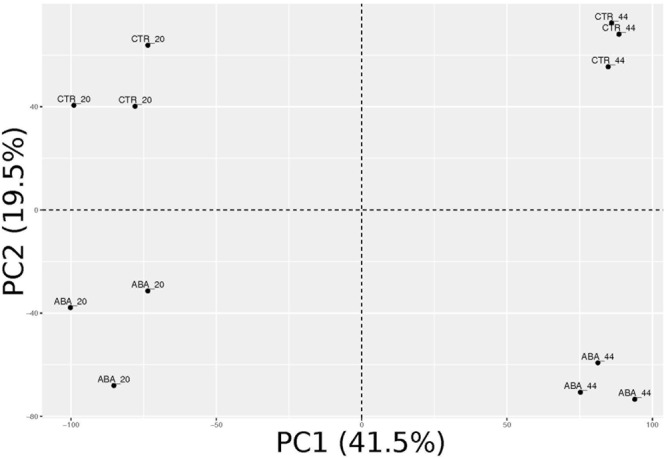
Principal component analysis (PCA) of ABA-treated and control berry skin samples transcriptomes. Biological replicates are grouped and very well separated according to the experimental condition along the first two principal components, which together explain 61% of the overall variance.

Transcriptomes of treated and control samples were analyzed by RNA-sequencing (Supplementary Table S2). Principal component analysis (PCA, **Figure 3**) shows that the four conditions are well-separated, while the biological replicates are grouped together. Major variance (41.5%) distinguished the two time-points (20 vs. 44 h), while treated vs. untreated berries were neatly separated along the second principal component, which explained 19.5% of the variance. It appeared that ABA treatment extensively impacted on pre-véraison berry skin transcriptome.

**FIGURE 4:**
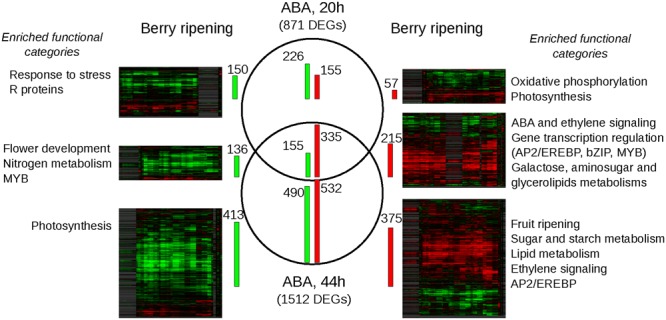
Comparison between the ABA-differentially expressed genes (DEGs) and their modulation during ripening analyzed using the grapevine compendium Vespucci. In the middle, Venn’s diagram of the DEGs between treated vs. control samples at 20 and 44 h, obtained combining statistical significance (*t*-test analysis *p*-value < 0.05) and twofold change threshold. Green bars on the left are proportional to the number of repressed genes, while red bars on the right to the number of induced genes. On left and right sides, heatmaps of the down- and up-ABA modulated genes obtained in Vespucci, selecting 7 experiments on berry ripening performed in six *Vitis vinifera* cv.: Cabernet Sauvignon (GSE11406), Sauvignon Blanc (GSE34634), Corvina (GSE36128), Pinot Noir (GSE49569 and GSE31674), Muscat Hamburg (GSE41206) and Norton (GSE24561). Genes are clustered according to their expression profiles. The number of the transcripts coherently modulated in ABA treatment and berry ripening is indicated beside the heatmaps by green/red bars. Outer left and right columns, enriched functional categories of each subset of genes coherently modulated by ABA and during ripening are reported. For the complete output of the GO and Vitisnet enrichment analyses, refer to Supplementary Table S4.

Treated vs. untreated samples within each time-point were statistically compared to extract the lists of significantly modulated genes and a further restriction on fold change (greater than 2) was applied. 871 genes resulted modulated at 20 h by ABA and 1512 at 44 h; 490 genes were modulated at both time-points, with a coherent trend (**Figure 4**; and Supplementary Table S3). Interestingly, ABA-induced transcriptional modulation increased over time, regardless of the absence of the external stimulus. This amplification in the response suggests that ABA likely acted as a primer of a broad cellular program. According to RNA-seq analysis, NCED1 was induced 32 and 64 times at 20 and 44 h, respectively, whereas LOXA was induced 3 and 12 times, thus confirming previous qPCR analysis. Genes have been functionally annotated using Vitisnet (Grimplet et al., 2009) integrated with manual curation. For functional class enrichment analyses both Vitisnet and Gene Ontology were used (Supplementary Table S4). Genes modulated exclusively at 20 h, the less abundant group, were enriched in classes related to stress, cell wall modification, photosynthesis, respiration and translation. They could represent a stress response due to the excess or sudden delivery of ABA in the treatment, which induced a high turn-over of proteins involved in basic energy metabolism and cell wall. The set of ABA-positively modulated genes at both 20 and 44 h was enriched in genes involved in cell regulation: ABA and ethylene networks were over-represented, along with transcription factors related to these hormones, such as members of the large bZIP, APETALA2 and MYB families. At 44 h post-treatment, the number of genes positively modulated equaled that of the negatively modulated and the functional categories related to the metabolic pathways typical of the ripening process were enriched. Lipid and carbohydrate metabolism, cell wall modification, and flavonoid metabolism were over-represented among the up-regulated genes, supporting the role ABA plays in regulation of sugar metabolism and accumulation, cell enlargement and softening and color development. Ethylene, Auxin and ABA-related categories remained over-represented, suggesting that these hormones regulated not only the onset but also the process of grape berry ripening. Photosynthesis was over-represented among the down-regulated genes, suggesting that ABA triggers the switching off of this basal metabolism inducing the transition to a specialized sink organ, such as the ripe berry.

### Most of ABA Responsive Genes Are Involved in Ripening

In order to outline the role of ABA at ripening onset, a meta-analysis using the grapevine expression data compendium Vespucci (Moretto et al., 2016) was performed. Seven experiments in which berry ripening transcriptome had been analyzed were selected to visualize how the sets of genes modulated in the skin by ABA at the two time-points were modulated during physiological ripening. These experiments were performed in different berry tissues (seed, pulp, skin, and pericarp) in six different cultivars: Cabernet Sauvignon (GSE11406), Sauvignon Blanc (GSE34634), Corvina (GSE36128), Pinot Noir (GSE49569 and GSE31674), Muscat Hamburg (GSE41206) and Norton (GSE24561). Heatmaps representing the comparisons are shown in **Figure 4**, while the tables which generated the heatmaps, downloaded from Vespucci website, are available as Supplementary Table S5. Within each comparison, a percentage of genes ranging between 49 and 87% were modulated coherently by ABA and during ripening in all tissues and cultivars (**Figure 4**). However, the heatmaps showed also variable profiles, likely related to the tissue and/or cultivar specific modulation of these transcripts. While the smallest overlap occurred with genes up-modulated exclusively at 20 h, the sets of genes modulated at both 20 and 44 h and those modulated only after 44 h showed a more extended overlap with ripening, between 64 and 87%, higher when considering the down-regulated genes. Functional categories enrichment analyses were repeated on the restricted sets of genes modulated both by ABA and during ripening (**Figure 4** and Supplementary Table S4). In general, the analysis reproduced the results described in the previous section, with small refinements, such as for genes up-modulated at 20 and 44 h in which more specific categories related to carbohydrate metabolism, such as aminosugars, galactose and glycerolipids, appeared enriched or changes, as occurred for genes up-modulated at 44 h, in which the two categories of cell wall and flavonoid biosynthesis were lost while that of fruit ripening and abscission were gained. On a broad scale, we show that green hard berries treated with ABA are not simply responding to a stimulus, rather activating genes that are typical of the ripening program.

Palumbo and collaborators highlighted that the transition from immature-to-mature stage in the berry is characterized by an extensive transcriptomic down-regulation, anti-correlated to a small group of 190 so called “switch genes” (Palumbo et al., 2014). Interestingly, we identified in our set of positively modulated genes 80 of such “switch genes” (Supplementary Table S6). This finding is consistent with ABA playing an important role in ripening regulation, partly by switching off typical vegetative pathways, such as those related to photosynthesis. Besides, 13 out of these 80 candidate “ABA-responsive switch genes” were predicted to be regulated post-transcriptionally by miRNA (Palumbo et al., 2014), suggesting that ABA modulation can occur both via direct targets activation and via post-transcriptional mechanisms.

Finally, we compared our results with the list of genes modulated in a grapevine cell culture by a 1-h treatment with 20 μM ABA (Nicolas et al., 2014). Only 55 genes were modulated by ABA in both experiments and they were mostly up-regulated (Supplementary Table S7). They are related to ABA network and cell response to abiotic stresses, such as drought, dehydration, osmotic stress and potentially represent a basal ABA signaling core conserved in any type of cell.

### ABA Network in Berry Skin at the Onset of Ripening

**FIGURE 5:**
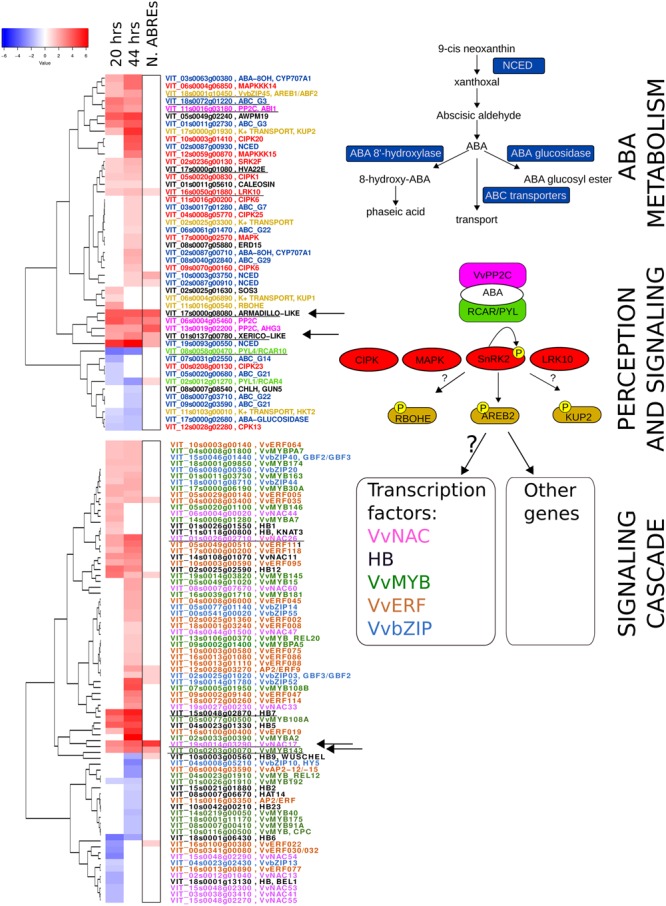
Abscisic acid network in the berry skin at ripening onset. On the right, from top to bottom, known gene families involved in ABA metabolism (blue), perception (light green and pink), signaling cascade activation (red) and down-stream regulators, such as target of kinases phosphorylation (light brown) and five transcription factor families are depicted. On the left, two heatmaps show the ABA modulation at 20 and 44 h of the genes belonging to the indicated gene families (same color). The boxed column shows the information on the number of ABREs in the promoter region of these genes (from 0 up to 4), coming from *in silico* promoter analysis. Underlined genes are modulated in the same way in grape berry cell cultures treated with ABA, reported in Nicolas et al. (2014). Arrows indicate the genes chosen for *in vivo* promoter activation assay by VvABF2.

Abscisic acid treatment of berries at pre-véraison stage significantly modulated several genes involved in ABA metabolism, perception and signaling, which are summarized in **Figure 5**. All the gene families involved in ABA metabolism appeared transcriptionally affected by ABA: NCED family, involved in ABA biosynthesis, ABA 8′ hydroxylase, in its degradation, ABA glucosidase, in ABA conjugation with sugar moieties and ABC transporters of the G subfamily, involved in ABA transport. The participation of the different NCED isoforms in berry ripening has been widely reported (Lund et al., 2008; Wheeler et al., 2009; Sun et al., 2010; Young et al., 2012). Using Vespucci, we could visualize the expression of the five NCEDs present in grapevine genome in at least three cultivars during berry development and post-harvest withering (**Supplementary Figure S2**). By combining this information with our results, we can state that VIT_19s0093g00550, called NCED3 in Young et al. (2012) and NCED1 in Sun et al. (2010), is very rapidly induced in the skin by ABA treatment and its upregulation specifically occurs in the pulp and skin tissues at véraison, while the gene is not modulated later in ripening (**Supplementary Figure S2**). In our results, there are two other NCEDs which are modulated though to a lesser extent and only at 44 h: VIT_02s0087g00910, which is also modulated during post-harvest withering in Corvina, and VIT_10s0003g03750, which does not seem related to the process of berry ripening (**Supplementary Figure S2**). To understand their function, further investigations are needed. Instead, from Vespucci analysis, the isoform VIT_02s0087g00930 seems induced during the whole berry ripening in all tissues, slightly more in the pulp.

Concerning ABA perception and signaling, both ABA receptors of the PYL/PYR/RCAR family and PP2C phosphatases were affected by the presence of exogenous ABA. The two receptors (VIT_08s0058g00470 and VIT_02s0012g01270), previously identified as RCAR5 and 7 by Boneh et al. (2011) and as PYL3 and PYL1 by Li et al. (2012), were down-regulated in our experiment. Three PP2C phosphatases were strongly induced at 20 and 44 h: VIT_11s0016g03180 was identified as AtABI1 homolog by phylogenetic analysis (named PP2C-2 in Gambetta et al., 2010), and was characterized in leaf and root (named PP2C4) by Boneh et al. (2011). VIT_06s0004g05460 and VIT_13s0019g02200, corresponding to PP2C-6 and PP2C-3 in Gambetta et al. (2010) and PP2C9 and PP2C8 in Boneh et al. (2011) were found induced at ripening onset in developing berries, anticipated in deficit irrigation conditions and induced by ABA in the skin of *in vitro* cultured berries. Out of the five identified Snf1-related kinases involved in ABA response, one (VIT_02s0236g00130) corresponding to SnRK2.1 in Boneh et al. (2012) was up-regulated at 20 and 44 h. In **Figure 5**, other kinases belonging to the calcium dependent protein kinases family (CPK/CDPK/CDKs) or calcineurin B-like interacting protein kinases (CIPKs) and the three tiers of mitogen-activated protein kinase cascades (MAPKs, MAPKKs, and MAPKKKs) were included, based on Arabidopsis literature reporting their potential involvement in ABA signaling (reviewed in Finkelstein, 2013). In grapevine, some CPK and CDPK have been studied due to their up-regulation at véraison and relationship to ABA or drought stress (Yu et al., 2006; Cuéllar et al., 2013). Interestingly, also a LRK10 receptor kinase was found induced at 20 and 44 h and was included, as it is induced also in ABA-treated cell culture and in Arabidopsis one isoform, AtLRK10L1.2, is implicated in ABA response and drought resistance (Lim et al., 2015).

Known direct targets of SnRK2 phosphorylation are NADPH oxidases of the Respiratory Burst Oxidase family (Rboh), leading to the production of hydrogen peroxide, plasma membrane anion and K+-channels, regulating ion transport and stomata opening, and basic domain/leucine zipper (bZIP) transcription factors of the ABA responsive element binding factor subgroup (AREB/ABF), mediating ABA-responsive genes transcription (Wang P. et al., 2013). In our experiment, representatives for all these functional categories were modulated (**Figure 5**, top). Interestingly, only one member of the grapevine AREB/ABF predicted subgroup (Liu et al., 2014) was modulated, namely the VvAREB/ABF2 (VvbZIP045, VIT_18s0001g10450), recently characterized by Nicolas et al. (2014). VvAREB/ABF2 likely represents the isoform phosphorylated by SnRK2 in berry skin cells at ripening onset and activating down-stream genes of the ABA signaling cascade.

### Extending the Regulatory Circuit of ABA Signaling

**FIGURE 6:**
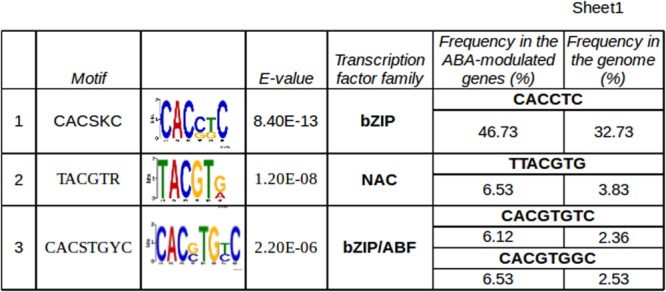
*In silico* promoter analysis of the genes modulated at both 20 and 44 h. The 1 kb promoter regions have been analyzed in DREME (Bailey, 2011) and motif enrichment has been calculated using the whole genome promoters as reference. The three most enriched motifs and the transcription factor families they are likely recognized by, are reported. A refined search of the most enriched exact sequence motifs has been performed using Patmatch (Yan et al., 2005) and the corresponding frequencies are reported in the last two columns.

With the aim of identifying candidate target genes of VvABF2 activity, promoter regions of the genes modulated at 20 and 44 h were analyzed to find significantly enriched *cis*-acting motifs. The three most enriched motifs that were found were recognized by bZIP transcription factors, NAC and ABF subgroup of the bZIP family, respectively (**Figure 6**). Calculating the frequency of refined consensus motifs highlighted that the ABRE motif, “CACGTGT/GC,” was about threefold more represented in our ABA-modulated gene set compared to the whole genome set of promoters. The recurrence of ABREs within each promoter was calculated: it ranged from 0 to 4 and is represented in **Figure 5**. ABREs are present in genes involved in ABA network, like NCEDs, PP2Cs and PYL/RCAR receptors but also in some TFs of the NAC, MYB, HB, bZIP and ERF families. In particular, VvMYB143 and VvNAC17 were the most enriched in ABREs and were also modulated by ABA in cell cultures (Nicolas et al., 2014). VvMYB143 belongs to the subgroup S11 of R2R3-MYB TFs, whose function has not been characterized yet (Wong et al., 2016). VvNAC17 belongs to subgroup III (Wang N. et al., 2013) and has been recently characterized for VvABF2 activation in tobacco protoplasts (Nicolas et al., 2014). Other two genes, VIT_17s0000g08080 and VIT_01s0137g00780, were highly modulated by ABA in our experiment and also in grapevine cell culture (Nicolas et al., 2014) and possess 4 and 2 ABREs in their promoters, respectively. The first gene is annotated as Armadillo b-catenin, because it contains the 3D motif Armadillo, involved in binding of large molecules such as proteins or DNA but also the U-box domain, for recruiting E2-adenylated ubiquitin in the protein degradation pathway (Coates, 2003). The gene VIT_01s0137g00780 was annotated as “unknown.” RNA-seq data have been used to refine this gene prediction, revealing an incorrect splicing site (Supplementary Table S8). The improved transcript prediction based on reads mapping was used for homology search by BLAST algorithm in other species, highlighting the presence of a RING Zinc finger domain, as in Xerico (Ko et al., 2006). Interestingly, in Arabidopsis, genes containing these domains are reported to be involved in ABA response modulation and in drought resistance (Ko et al., 2006; Moody et al., 2016). These two genes will be hereafter referred to as Armadillo-like and Xerico-like, for brevity. Vespucci analysis showed that VvNAC17 and Armadillo-like were part the genes modulated by ABA at 20 and 44 h and during ripening, while VvMYB143 and Xerico-like were characterized by more variable profiles in the different cultivars and tissues and would need more specific investigation to precisely describe their modulation.

**FIGURE 7:**
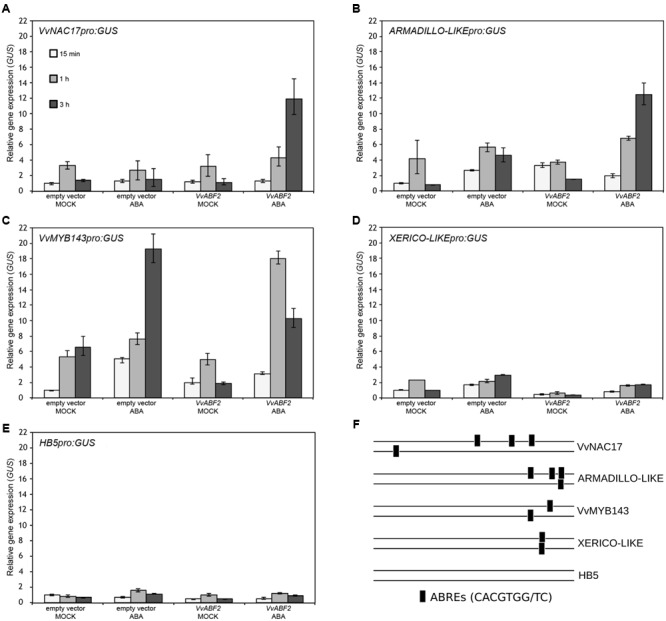
*Agrobacterium*-mediated transient *trans*-activation assay in *N. benthamiana*. qPCR analysis of GUS expression in leaves of *N. benthamiana* co-transfected with the activating construct CaMV35Spro:VvABF2 and the following target constructs: **(A)** VvNAC17pro:GUS, **(B)** ARMADILLO-likepro:GUS, **(C)** VvMYB143pro:GUS, **(D)** XERICO-likepro:GUS, **(E)** HB5pro:GUS. Leaves co-infiltrated with the pK7WG2 vector devoid of the VvABF2 gene (empty vector) were used as a negative control for the *trans*-activation assay. Forty-eight hours after the first infiltration, leaves were infiltrated with 50 μM ABA or with a mock solution and samples were collected after 15 min, 1, and 3 h. Relative GUS expression was determined using GUS-specific primers and normalized to the expression of the tobacco EF-1α gene. Data are means of four biological replicates ± SD. **(F)** Distribution of the ABRE motifs in the five 1 kb promoters analyzed.

These four genes were further investigated in a transient *trans*-activation assay in *N. benthamiana* to verify their dependence on VvABF2 activity. The transcription factor HB5, activated by ABA but lacking ABREs in its promoter, was included as a negative control. Tobacco leaves were co-infiltrated with the target promoters fused to the reporter GUS along with either the VvABF2 activator or the control empty vector. The constitutive over-expression of ABF2 on its own is insufficient to induce expression of the downstream target genes, as ABF2 activity involves the ABA-dependent phosphorylation of its N-terminal domain, as demonstrated in Arabidopsis for AtABF2 (Fujita et al., 2005). Consequently, 2 days after the agro-infiltration, leaves were re-infiltrated either with 50 μM ABA or with a mock solution. GUS gene expression was analyzed by qPCR at 15 min, 1, and 3 h after the ABA treatment (**Figures 7A–E**). The promoters of VvNAC17 and Armadillo-like showed a remarkable activation by VvABF2 following ABA treatment, in agreement with the presence of four ABREs in their sequences (**Figure 7F**). Expression of VvMYB143, which contains two ABRE motifs in its promoter, was activated by ABA, irrespectively of the presence of VvABF2. Conversely, despite the presence of two ABF binding domains, the Xerico-like promoter did not show any activation in all the conditions, as observed for the negative control gene HB5.

## Discussion

This work outlines the importance of ABA in the initial phases of berry skin ripening and describes in detail the molecular components of its network, discriminating ripening-specific isoforms and identifying new elements of the signaling cascade.

The positive correlation between the rate of ABA accumulation and the ripening rate of the berry has been firstly reported in 1973 (Coombe and Hale, 1973). Since then, numerous studies highlighted the reciprocal influence and importance of ABA, ethylene, auxins and brassinosteroids at the onset of ripening, based on the observation that variations in the level of an hormone affects the relative concentration of the others and consequently the timing of ripening (Coombe and Hale, 1973; Davies et al., 1997; Symons et al., 2006; Sun et al., 2010; Su et al., 2015). A previous work investigated at the transcriptomic level the effect of ABA on berry ripening, by means of the Affymetrix Vitis Genechip (Koyama et al., 2010). More recently two additional works, performed using NimbleGen whole genome arrays, addressed the transcriptional response to ABA in grapevine cell cultures and in different grapevine organs (Nicolas et al., 2014; Rattanakon et al., 2016). These studies highlighted that ABA is a ubiquitous signal raising specific responses according to the cell and tissue type and to the precise developmental stage. In the present work, great attention has been paid to the experimental setting, in the attempt to simulate the molecular events occurring in the berry just before ripening starts. Therefore, the precise developmental stage of green hard berry has been identified by means of a preliminary study in 2011, based on daily sampling during the 2 weeks before color break. This study suggested three molecular markers useful to define the pre-véraison stage, characterized by very low content of ABA and very low expression levels of the two genes LOXA and NCED, and the transition to the ripening stage, characterized by a significant increase in all these values.

In order to capture the early events of ABA response in the context of berry skin ripening onset regulation, green hard berries were collected in 2013 and treated with ABA for 20 h in liquid and then cultured for additional 24 h in the absence of exogenous ABA, to assay its role as a trigger. ABA uptake and its effect on berry development have been initially assayed by measuring the values of the three markers, which suggested the occurrence of the transition (**Figure 2**). Then berry skin transcriptomes have been analyzed by RNA-seq. PCA analysis highlighted that ABA treatment extensively impacted on berry skin cells transcriptome (**Figure 3**). In fact, 871 genes were modulated by ABA compared to mock treated samples after 20 h, and 1512 after 44 h, indicating that the response to ABA amplified over time, as a signaling cascade (**Figure 4**). Even though this cascade has been triggered by a small amount of exogenous ABA, we actually know from the up-regulation of the enzyme NCED and the measured intracellular ABA level at 44 h (**Figure 2**) that ABA triggered its own biosynthesis through a positive feedback loop. As our focus was on the role of ABA in ripening onset, we narrowed our attention on those genes modulated by ABA during physiological ripening. This comparison has been performed using the grapevine expression data compendium Vespucci (**Figure 4**). Remarkably, 1346 (71%) ABA-skin responsive genes appeared modulated also during ripening in the berry tissues and cultivars considered, strongly supporting the regulatory role this hormone has in this non-climacteric plant. Nonetheless, this analysis highlighted genes characterized by a more variable profile, related to tissue and/or cultivar specific modulation, which will deserve further characterization. Based on functional enrichment analyses, the categories related to signaling were over-represented within the genes induced at 20 and 44 h (**Figure 4** and Supplementary Table S4), namely the whole ABA network, from biosynthesis and perception to bZIP transcription factors and the ethylene signaling cascade mediated by AP2/EREBP transcription factors. It is interesting to note that some ethylene responsive factors were rapidly modulated by ABA before ethylene biosynthesis was stimulated, suggesting that they might actually represent points of convergence between the two hormones signaling cascades, explaining the tight interconnection between these two hormones at the onset of ripening (Sun et al., 2010). Even though not statistically over-represented, many genes involved in auxin response (IAA, SAUR, ARF), metabolism (GH3) and transport and five genes involved in brassinosteroids biosynthesis and signaling were modulated by ABA, supporting the previously proposed model of a complex integrated signaling network (Kuhn et al., 2014).

Conversely, functional classes related to ripening-specific metabolisms were found over-represented at the 44 h time-point. In particular, the up-regulated set was enriched in starch and sucrose metabolism, ethylene signaling, including AP2/EREBP transcription factors, whereas the down-modulated genes were mainly related to photosynthesis. The occurrence of this modulation at 44 h might reflect an indirect effect of ABA on these pathways, possibly mediated by other signals, such as ethylene and/or sugars. Besides, the modulation of many genes related to ions and water transport and cell wall modification supports the role of ABA in the fine tuning of sugar accumulation and water uptake to control cell osmosis which, in concert with cell wall structure modifications, drives the process of cell distension in the skin at the onset of berry ripening. The role of ABA in stimulating color accumulation through the activation of regulatory and structural genes of the anthocyanin pathway is still elusive. Treatments with this hormone after véraison were effective in stimulating berry coloring (Peppi et al., 2008; Ferrara et al., 2013; Katayama-Ikegami et al., 2016). Other studies showed that sugars were effective in promoting anthocyanins accumulation both in grapevine cell cultures (reviewed in Lecourieux et al., 2014) and *in vitro* berry cultures (Dai et al., 2014). In our analysis, we observed the up-regulation of the TF VvMYBA2 and of some structural genes such as flavonoid 3′5′ hydroxylases and UDP-glucose: anthocyanidin 5,3-O-glucosyltransferases, at 44 h, even though the whole pathway was not statistically enriched and we did not observe berry coloring. This delay was probably due to the fact that other cofactors from the MYB-bHLH-WD40 complex (such as MYC1 or MYCA1) were still not induced, suggesting that other signals beside ABA were required. Interestingly, VvMYBA7, recently characterized as a regulator of the anthocyanins synthesis in vegetative organs, was up-regulated by ABA but only at the 20 h time-point, suggesting its direct induction by ABA, but not its participation to the ripening program (Matus et al., 2017). The role of ABA in down-regulating photosynthesis under stressful conditions, such as drought, salinity or low temperature, and during developmental processes, such as senescence (Lee et al., 2004; Yang et al., 2011; Gao et al., 2016), has been extensively described. It is not surprising then that at 44 h the functional enriched categories within the down-regulated genes involve different aspects of the photosynthetic metabolism. The switching off of this metabolism, central in green vegetative tissues, requires a highly regulated and concerted ensemble of reactions in order to avoid dangerous accumulation of reactive oxygen species or metabolic unbalances. The comparison with a previous meta-analysis focused on the genes involved in the transition from immature-to-mature stage in the berries highlighted that a relevant proportion (42%) of these genes were up-regulated by ABA (Palumbo et al., 2014). This observation supports the importance of ABA in triggering the transition to ripening and the down-regulation of the photosynthetic metabolism (Supplementary Table S5). Moreover, this comparison indicated that ABA regulates gene expression not only at the transcriptional level, by means of transcription factors modulation, but also post-transcriptionally, modulating miRNAs transcription.

As mentioned above, ABA is an ancient and ubiquitous signaling molecule, evolutionary linked to plant adaptation to dry terrestrial land (Takezawa et al., 2011; Wanke, 2011). It controls transpiration, dehydration tolerance and other water status-associated processes such as seed and bud maturation and dormancy, root growth, leaf morphogenesis and senescence, thus making this compound a key player in integrating plants growth and development with environmental conditions (Nambara and Kuchitsu, 2011). The specificity of cellular ABA response must thus rely on specific isoforms of upstream perception components and on the wide variety of downstream signaling cascades. In this study, such ripening specific isoforms have been identified (**Figure 5**) and they represent strong candidates for experimental validation. Concerning the ABA biosynthetic enzyme NCED, a véraison specific isoform, VIT_19s0093g00550 was identified (**Supplementary Figure S2**). Two ABC transporters of the G subfamily, VIT_18s0072g01220 and VIT_01s0011g02730, could be involved in ABA import/export, the former being induced also in ABA-treated grapevine cell cultures (Nicolas et al., 2014). Three PP2C genes out of the nine previously characterized (Boneh et al., 2011), were up-regulated in the berry by ABA and during ripening; one of these, ABI1, is induced also in the study by (Nicolas et al., 2014). Many kinases of the calcium- or calcineurin-dependent families, beside the better characterized SnRK2, are induced by ABA and likely to be related to this signaling cascade. Known transcription factors directly activated by SnRK2 belong to the bZIP family and are named ABA responsive element binding (ABRE/ABF). In grapevine, VvABF2 (VIT_18s0001g10450, VvbZIP45), initially named GRIP55 due to its induction during ripening (Davies and Robinson, 2000), has been exhaustively characterized, showing its induction by ABA and its prevalent transcription in berry skin and seeds (Nicolas et al., 2014). As we also found VvABF2 induced by ABA in berry skins in our study, we tried to identify its possible targets. By an *in silico* promoter analysis of the genes modulated at 20 and 44 h, a motif very similar to the ABA responsive elements (ABREs) present in the Arabidopsis database was found as significantly enriched (**Figure 6**). We focused our attention on four genes that were strongly up-regulated by ABA at both time-points and in cell cultures and enriched in ABREs: two TFs, VvMYB143 and VvNAC17, and two uncharacterized genes possibly involved in proteasome-dependent protein degradation, Armadillo-like and Xerico-like. These genes are modulated during berry ripening, even if MYB143 and Xerico-like need further investigation as they show a tissue and/or cultivar specific behavior. Interestingly enough, all these genes are reported in literature to be related to osmotic stress (Denekamp and Smeekens, 2003), drought stress (Ko et al., 2006) and/or ABA accumulation (Kong et al., 2015). In particular, the transcriptional modulation of Armadillo-like and Xerico-like genes and their involvement in ABA response via protein stability indicates a third level of ABA response regulation, in addition to the transcriptional and miRNA-mediated post-transcriptional ones. This translational regulation level could affect either the perception/activation mechanism of ABA signaling, as suggested by Kong et al. (2015) or proteins down-stream in the cascade, as suggested by Liu et al. (2011) and Seo et al. (2012) in Arabidopsis.

The trans-activation assay performed in tobacco leaves showed that VvNAC17 and Armadillo-like were strongly activated by VvABF2, consistently with the presence of four ABREs in their promoters (**Figure 7**). Instead, VvMYB143 was activated by ABA irrespectively of the presence of VvABF2, suggesting that endogenous tobacco transcription factors can mediate its ABA dependent expression. Finally, Xerico-like neither showed significant activation by ABA nor by VvABF2. This is in apparent contrast with the observation that Xerico-like expression is activated in ABA-treated grapevine cell cultures constitutively over-expressing VvABF2 (Nicolas et al., 2014). One possible explanation is that other possibly grape-specific factors are required to prime Xerico-like expression in addition to VvABF2 (e.g., additional transcription factors or VvABF2-interacting proteins). Interestingly, an *in silico* analysis identified Xerico-like among the co-expressed VvMYB143 genes and enriched in MYB-core type I binding motif, suggesting that VvMYB143 might be the regulator of Xerico-like (Wong et al., 2016).

## Conclusion

We proved the importance of ABA signaling to trigger the onset of ripening in the skin of green hard berries, occurring via an extensive gene modulation. Many molecular components of the ABA network, encompassing metabolism, perception and signaling, have been identified and many have been proposed as candidates to be experimentally validated. Four genes have been experimentally characterized showing different behaviors in response to ABA. As these genes are related to ABA/drought tolerance in other species, it will be of interest to functionally characterize them not only at ripening onset, but also under abiotic stress conditions.

## Author Contributions

SP designed the study, performed time-course study in 2011 and ABA treatment in 2013 together with DB, supervised all the analyses and drafted the manuscript, GB performed the RNAseq experiment and worked on data analysis, KE, PS, and MM performed the bioinformatic analyses (RNAseq raw data analysis, gene co-expression and promoter analyses), GC, LS, and MG performed promoter activation assays in tobacco leaves, GG did ABA and lipid peroxidation analyses, CT revised the manuscript, CM contributed to the design of the study and interpretation of the results, supervised all the study and revised the manuscript. All authors revised and approved the final manuscript.

## Conflict of Interest Statement

The authors declare that the research was conducted in the absence of any commercial or financial relationships that could be construed as a potential conflict of interest.

## Footnotes

1 https://www.bioinformatics.babraham.ac.uk/projects/fastqc/

2 http://genomes.cribi.unipd.it/

3 https://github.com/onertipaday/ShinyVolcanoPlot

4 www.ncbi.nlm.nih.gov/sra

5 http://genomes.cribi.unipd.it/DATA/V1/ANNOTATION/

## Supplementary Material

FIGURE S1 Description of the ABA treatment.

FIGURE S2 Visualization of NCED gene family during berry ripening in three *Vitis vinifera* cultivars.

## Abbreviations

ABA: abscisic acid
ABRE: ABA-responsive element
AREB/ABF: ABRE-binding proteins/ABRE-binding factors
CIPK: CBL-interacting protein kinase
CPK: calcium-dependent protein kinase
NCED: 9-*cis*-epoxycarotenoid dioxygenase
PP2C: protein phosphatase 2C
PYR/PYL/RCAR: pyrabactin resistance1/PYR1-like/regulatory components of ABA receptors
RBOH: respiratory burst oxidase homolog
SnRK2: sucrose-non-fermenting1-related kinase 2

## References

[B1] Alexa A. Rahnenfuhrer J. (2016). *topGO: Enrichment Analysis for Gene Ontology. R package Version 2.28.0*. Available at: https://www.bioconductor.org/

[B2] Antolìn M. C. Baigorri H. De Luis I. Aguirrezàbak F. Geny L. Broquedis M. (2003). ABA during reproductive development in non-irrigated grapevines (*Vitis vinifera* L. cv. Tempranillo). *Aust. J. Grape Wine Res.* 9 169–176. 10.1111/j.1755-0238.2003.tb00266.x

[B3] Bailey T. L. (2011). DREME: motif discovery in transcription factor ChIP-seq data. *Bioinformatics* 27 1653–1659. 10.1093/bioinformatics/btr261 21543442 PMC3106199

[B4] Bastías A. Yañez M. Osorio S. Arbona V. Gómez-Cadenas A. Fernie A. R. (2014). The transcription factor AREB1 regulates primary metabolic pathways in tomato fruits. *J. Exp. Bot.* 65 2351–2363. 10.1093/jxb/eru114 24659489 PMC4036503

[B5] Boneh U. Biton I. Schwartz A. Ben-Ari G. (2012). Characterization of the ABA signal transduction pathway in *Vitis vinifera*. *Plant Sci.* 187 89–96. 10.1016/j.plantsci.2012.01.015 22404836

[B6] Boneh U. Biton I. Zheng C. Schwartz A. Ben-Ari G. (2011). Characterization of potential ABA receptors in *Vitis vinifera*. *Plant Cell Rep.* 31 311–321. 10.1007/s00299-011-1166-z 22016084

[B7] Castellarin S. D. Gambetta G. A. Wada H. Krasnow M. N. Cramer G. R. Peterlunger E. (2015). Characterization of major ripening events during softening in grape: turgor, sugar accumulation, abscisic acid metabolism, colour development, and their relationship with growth. *J. Exp. Bot.* 67 709–722. 10.1093/jxb/erv483 26590311 PMC4737070

[B8] Castellarin S. D. Gambetta G. A. Wada H. Shackel K. A. Matthews M. A. (2011). Fruit ripening in *Vitis vinifera*: spatiotemporal relationships among turgor, sugar accumulation, and anthocyanin biosynthesis. *J. Exp. Bot.* 62 4345–4354. 10.1093/jxb/err150 21586429 PMC3153685

[B9] Castellarin S. D. Pfeiffer A. Sivilotti P. Degan M. Peterlunger E. Di Gaspero G. (2007). Transcriptional regulation of anthocyanin biosynthesis in ripening fruits of grapevine under seasonal water deficit. *Plant Cell Environ.* 30 1381–1399. 10.1111/j.1365-3040.2007.01716.x 17897409

[B10] Cheng G. W. Breen P. J. (1991). Activity of Phenylalanine Ammonia-Lyase (PAL) and concentrations of anthocyanins and phenolics in developing strawberry fruit. *J. Am. Soc. Hortic. Sci.* 116 865–869.

[B11] Coates J. C. (2003). Armadillo repeat proteins: beyond the animal kingdom. *Trends Cell Biol.* 13 463–471. 10.1016/S0962-8924(03)00167-3 12946625

[B12] Coombe B. G. Hale C. R. (1973). The hormone content of ripening grape berries and the effects of growth substance treatments. *Plant Physiol.* 51 629–634. 10.1104/pp.51.4.629 16658384 PMC366320

[B13] Cuéllar T. Azeem F. Andrianteranagna M. Pascaud F. Verdeil J.-L. Sentenac H. (2013). Potassium transport in developing fleshy fruits: the grapevine inward K(+) channel VvK1.2 is activated by CIPK-CBL complexes and induced in ripening berry flesh cells. *Plant J.* 73 1006–1018. 10.1111/tpj.12092 23217029

[B14] Dai Z. W. Meddar M. Renaud C. Merlin I. Hilbert G. Delrot S. (2014). Long-term in vitro culture of grape berries and its application to assess the effects of sugar supply on anthocyanin accumulation. *J. Exp. Bot.* 65 4665–4677. 10.1093/jxb/ert489 24477640 PMC4115254

[B15] Davies C. Boss P. K. Robinson S. P. (1997). Treatment of grape berries, a nonclimacteric fruit with a synthetic auxin, retards ripening and alters the expression of developmentally regulated genes. *Plant Physiol.* 115 1155–1161. 10.1104/PP.115.3.1155 12223864 PMC158580

[B16] Davies C. Robinson S. P. (2000). Differential screening indicates a dramatic change in mRNA profiles during grape berry ripening. Cloning and characterization of cDNAs encoding putative cell wall and stress response proteins. *Plant Physiol.* 122 803–812. 10712544 10.1104/pp.122.3.803 PMC58916

[B17] Deluc L. G. Grimplet J. Wheatley M. D. Tillett R. L. Quilici D. R. Osborne C. (2007). Transcriptomic and metabolite analyses of Cabernet Sauvignon grape berry development. *BMC Genomics* 8:429 10.1186/1471-2164-8-429 PMC2220006 18034876

[B18] Denekamp M. Smeekens S. C. (2003). Integration of wounding and osmotic stress signals determines the expression of the AtMYB102 transcription factor gene. *Plant Physiol.* 132 1415–1423. 10.1104/PP.102.019273 12857823 PMC167081

[B19] Fasoli M. Dal Santo S. Zenoni S. Tornielli G. B. Farina L. Zamboni A. (2012). The grapevine expression atlas reveals a deep transcriptome shift driving the entire plant into a maturation program. *Plant Cell* 24 3489–3505. 10.1105/tpc.112.100230 22948079 PMC3480284

[B20] Ferrandino A. Lovisolo C. (2014). Abiotic stress effects on grapevine (*Vitis vinifera* L.): Focus on abscisic acid-mediated consequences on secondary metabolism and berry quality. *Environ. Exp. Bot.* 103 138–147. 10.1016/j.envexpbot.2013.10.012

[B21] Ferrara G. Mazzeo A. Matarrese A. M. S. Pacucci C. Pacifico A. Gambacorta G. (2013). Application of abscisic acid (S-ABA) to “crimson seedless” grape berries in a Mediterranean climate: effects on color, chemical characteristics, metabolic profile, and S-ABA Concentration. *J. Plant Growth Regul.* 32 491–505. 10.1007/s00344-012-9316-2

[B22] Finkelstein R. (2013). Abscisic acid synthesis and response. *Arabidopsis Book* 11:e0166 10.1199/tab.0166 PMC3833200 24273463

[B23] Fujita Y. Fujita M. Satoh R. Maruyama K. Parvez M. M. Seki M. (2005). AREB1 is a transcription activator of novel ABRE-dependent ABA signaling that enhances drought stress tolerance in *Arabidopsis*. *Plant Cell* 17 3470–3488. 10.1105/tpc.105.035659 16284313 PMC1315382

[B24] Gambetta G. A. Matthews M. A. Shaghasi T. H. McElrone A. J. Castellarin S. D. (2010). Sugar and abscisic acid signaling orthologs are activated at the onset of ripening in grape. *Planta* 232 219–234. 10.1007/s00425-010-1165-2 20407788 PMC2872022

[B25] Gao S. Gao J. Zhu X. Song Y. Li Z. Ren G. (2016). ABF2, ABF3, and ABF4 promote ABA-mediated chlorophyll degradation and leaf senescence by transcriptional activation of chlorophyll catabolic genes and senescence-associated genes in *Arabidopsis*. *Mol. Plant* 9 1272–1285. 10.1016/j.molp.2016.06.006 27373216

[B26] Grimplet J. Cramer G. R. Dickerson J. A. Mathiason K. Van Hemert J. Fennell A. Y. (2009). VitisNet: “omics” integration through grapevine molecular networks. *PLoS ONE* 4:e8365 10.1371/journal.pone.0008365 PMC2791446 20027228

[B27] Jaillon O. Aury J.-M. Noel B. Policriti A. Clepet C. Casagrande A. (2007). The grapevine genome sequence suggests ancestral hexaploidization in major angiosperm phyla. *Nature* 449 463–467. 10.1038/nature06148 17721507

[B28] Jarzyniak K. M. Jasinski M. (2014). Membrane transporters and drought resistance - a complex issue. *Front. Plant Sci.* 5:687 10.3389/fpls.2014.00687 PMC4255493 25538721

[B29] Karimi M. Inzé D. Depicker A. (2002). GATEWAYTM vectors for *Agrobacterium*-mediated plant transformation. *Trends Plant Sci.* 7 193–195. 10.1016/S1360-1385(02)02251-3 11992820

[B30] Katayama-Ikegami A. Sakamoto T. Shibuya K. Katayama T. Gao-Takai M. (2016). Effects of abscisic acid treatment on berry coloration and expression of flavonoid biosynthesis genes in grape. *Am. J. Plant Sci.* 7 1325–1336. 10.4236/ajps.2016.79127

[B31] Ko J.-H. Yang S. H. Han K.-H. (2006). Upregulation of an *Arabidopsis* RING-H2 gene, XERICO, confers drought tolerance through increased abscisic acid biosynthesis. *Plant J.* 47 343–355. 10.1111/j.1365-313X.2006.02782.x 16792696

[B32] Kondo S. Kawai M. (1998). Relationship between free and conjugated ABA levels in seeded and gibberellin-treated seedless, maturing “pione” graape berries. *J. Am. Soc. Hort. Sci.* 123 750–754.

[B33] Kong L. Cheng J. Zhu Y. Ding Y. Meng J. Chen Z. (2015). Degradation of the ABA co-receptor ABI1 by PUB12/13 U-box E3 ligases. *Nat. Commun.* 6:8630 10.1038/ncomms9630 PMC4667695 26482222

[B34] Koyama K. Sadamatsu K. Goto-Yamamoto N. (2010). Abscisic acid stimulated ripening and gene expression in berry skins of the Cabernet Sauvignon grape. *Funct. Integr. Genomics* 10 367–381. 10.1007/s10142-009-0145-8 19841954

[B35] Kuhn N. Guan L. Dai Z. W. Wu B.-H. Lauvergeat V. Gomès E. (2014). Berry ripening: recently heard through the grapevine. *J. Exp. Bot.* 65 4543–4559. 10.1093/jxb/ert395 24285825

[B36] Law C. W. Chen Y. Shi W. Smyth G. K. (2014). voom: precision weights unlock linear model analysis tools for RNA-seq read counts. *Genome Biol.* 15:R29 10.1186/gb-2014-15-2-r29 PMC4053721 24485249

[B37] Lecourieux F. Kappel C. Lecourieux D. Serrano A. Torres E. Arce-Johnson P. (2014). An update on sugar transport and signalling in grapevine. *J. Exp. Bot.* 65 821–832. 10.1093/jxb/ert394 24323501

[B38] Lee R. H. Lin M. C. Chen S. C. (2004). A novel alkaline alpha-galactosidase gene is involved in rice leaf senescence. *Plant Mol. Biol.* 55 281–295. 15604681 10.1007/s11103-004-0641-0

[B39] Li G. Xin H. Zheng X. F. Li S. Hu Z. (2012). Identification of the abscisic acid receptor VvPYL1 in *Vitis vinifera*. *Plant Biol.* 14 244–248. 10.1111/j.1438-8677.2011.00504.x 21974741

[B40] Li X. (2011). Infiltration of *Nicotiana benthamiana* protocol for transient expression via *Agrobacterium*. *Bio-protocol* 1:e95 10.21769/BioProtoc.95

[B41] Liao Y. Smyth G. K. Shi W. (2013). The Subread aligner: fast, accurate and scalable read mapping by seed-and-vote. *Nucleic Acids Res.* 41:e108 10.1093/nar/gkt214 PMC3664803 23558742

[B42] Liao Y. Smyth G. K. Shi W. (2014). featureCounts: an efficient general purpose program for assigning sequence reads to genomic features. *Bioinformatics* 30 923–930. 10.1093/bioinformatics/btt656 24227677

[B43] Licausi F. Giorgi F. M. Zenoni S. Osti F. Pezzotti M. Perata P. (2010). Genomic and transcriptomic analysis of the AP2/ERF superfamily in *Vitis vinifera*. *BMC Genomics* 11:719 10.1186/1471-2164-11-719 PMC3022922 21171999

[B44] Lijavetzky D. Carbonell-Bejerano P. Grimplet J. Bravo G. Flores P. Fenoll J. (2012). Berry flesh and skin ripening features in *Vitis vinifera* as assessed by transcriptional profiling. *PLoS ONE* 7:e39547 10.1371/journal.pone.0039547 PMC3386993 22768087

[B45] Lim C. W. Yang S. H. Shin K. H. Lee S. C. Kim S. H. (2015). The AtLRK10L1.2, *Arabidopsis* ortholog of wheat LRK10, is involved in ABA-mediated signaling and drought resistance. *Plant Cell Rep.* 34 447–455. 10.1007/s00299-014-1724-2 25533478

[B46] Liu J. Chen N. Chen F. Cai B. Dal Santo S. Tornielli G. B. (2014). Genome-wide analysis and expression profile of the bZIP transcription factor gene family in grapevine (*Vitis vinifera*). *BMC Genomics* 15:281 10.1186/1471-2164-15-281 PMC4023599 24725365

[B47] Liu Y.-C. Wu Y.-R. Huang X.-H. Sun J. Xie Q. (2011). AtPUB19, a U-box E3 ubiquitin ligase, negatively regulates abscisic acid and drought responses in *Arabidopsis thaliana*. *Mol. Plant* 4 938–946. 10.1093/mp/ssr030 21502661 PMC3221247

[B48] Lund S. Peng F. Nayar T. Reid K. Schlosser J. (2008). Gene expression analyses in individual grape (*Vitis vinifera* L.) berries during ripening initiation reveal that pigmentation intensity is a valid indicator of developmental staging within the cluster. *Plant Mol. Biol.* 68 301–315. 10.1007/s11103-008-9371-z 18642093

[B49] Martin M. (2011). Cutadapt removes adapter sequences from high-throughput sequencing reads. *EMBnet.journal* 17 10–12.

[B50] Matus J. T. Cavallini E. Loyola R. Höll J. Finezzo L. Dal Santo S. (2017). A group of grapevine MYBA transcription factors located in chromosome 14 control anthocyanin synthesis in vegetative organs with different specificities compared to the berry color locus. *Plant J.* 5 1–17. 10.1111/TPJ.13558 28370629

[B51] Merlot S. Gosti F. Guerrier D. Vavasseur A. Giraudat J. (2001). The ABI1 and ABI2 protein phosphatases 2C act in a negative feedback regulatory loop of the abscisic acid signalling pathway. *Plant J.* 25 295–303. 10.1046/j.1365-313x.2001.00965.x 11208021

[B52] Moody L. A. Saidi Y. Gibbs D. J. Choudhary A. Holloway D. Vesty E. F. (2016). An ancient and conserved function for Armadillo-related proteins in the control of spore and seed germination by abscisic acid. *New Phytol.* 211 940–951. 10.1111/nph.13938 27040616 PMC4982054

[B53] Moretto M. Sonego P. Pilati S. Malacarne G. Costantini L. Grzeskowiak L. (2016). VESPUCCI: exploring patterns of gene expression in grapevine. *Front. Plant Sci.* 7:633 10.3389/fpls.2016.00633 PMC4862315 27242836

[B54] Nambara E. Kuchitsu K. (2011). Opening a new era of ABA research. *J. Plant Res.* 124 431–435. 10.1007/s10265-011-0437-7 21698351

[B55] Nambara E. Marion-Poll A. (2005). Abscisic acid biosynthesis and catabolism. *Annu. Rev. Plant Biol.* 56 165–185. 15862093 10.1146/annurev.arplant.56.032604.144046

[B56] Nicolas P. Lecourieux D. Kappel C. Cluzet S. Cramer G. Delrot S. (2014). The basic leucine zipper transcription factor ABSCISIC ACID RESPONSE ELEMENT-BINDING FACTOR2 is an important transcriptional regulator of abscisic acid-dependent grape berry ripening processes. *Plant Physiol.* 164 365–383. 10.1104/pp.113.231977 24276949 PMC3875815

[B57] O’Malley R. C. Huang S. C. Song L. Lewsey M. G. Bartlett A. Nery J. R. (2016). Cistrome and epicistrome features shape the regulatory DNA landscape. *Cell* 165 1280–1292. 10.1016/j.cell.2016.04.038 27203113 PMC4907330

[B58] Palumbo M. C. Zenoni S. Fasoli M. Massonnet M. Farina L. Castiglione F. (2014). Integrated network analysis identifies fight-club nodes as a class of hubs encompassing key putative switch genes that induce major transcriptome reprogramming during grapevine development. *Plant Cell* 26 4617–4635. 10.1105/tpc.114.133710 25490918 PMC4311215

[B59] Peppi M. C. Walker M. A. Fidelibus M. W. (2008). Application of abscisic acid rapidly upregulated UFGT gene expression and improved color of grape berries. *Vitis J. Grapevine Res.* 47 11–14.

[B60] Pfaffl M. W. (2001). A new mathematical model for relative quantification in real-time RT–PCR. *Nucleic Acids Res.* 29:e45. 10.1093/nar/29.9.e45 PMC55695 11328886

[B61] Pilati S. Brazzale D. Guella G. Milli A. Ruberti C. Biasioli F. (2014). The onset of grapevine berry ripening is characterized by ROS accumulation and lipoxygenase-mediated membrane peroxidation in the skin. *BMC Plant Biol.* 14:87 10.1186/1471-2229-14-87 PMC4021102 24693871

[B62] Pilati S. Perazzolli M. Malossini A. Cestaro A. Demattè L. Fontana P. (2007). Genome-wide transcriptional analysis of grapevine berry ripening reveals a set of genes similarly modulated during three seasons and the occurrence of an oxidative burst at vèraison. *BMC Genomics* 8:428 10.1186/1471-2164-8-428 PMC2228314 18034875

[B63] Rattanakon S. Ghan R. Gambetta G. A. Deluc L. G. Schlauch K. A. Cramer G. R. (2016). Abscisic acid transcriptomic signaling varies with grapevine organ. *BMC Plant Biol.* 16:72 10.1186/s12870-016-0763-y PMC4802729 27001301

[B64] Ruijter J. M. Ramakers C. Hoogaars W. M. H. Karlen Y. Bakker O. van den Hoff M. J. B. (2009). Amplification efficiency: linking baseline and bias in the analysis of quantitative PCR data. *Nucleic Acids Res.* 37:e45 10.1093/nar/gkp045 PMC2665230 19237396

[B65] Schmidt G. W. Delaney S. K. (2010). Stable internal reference genes for normalization of real-time RT-PCR in tobacco (*Nicotiana tabacum*) during development and abiotic stress. *Mol. Genet. Genomics* 283 233–241. 10.1007/s00438-010-0511-1 20098998

[B66] Seo D. H. Ryu M. Y. Jammes F. Hwang J. H. Turek M. Kang B. G. (2012). Roles of four Arabidopsis U-box E3 ubiquitin ligases in negative regulation of abscisic acid-mediated drought stress responses. *Plant Physiol.* 160 556–568. 10.1104/pp.112.202143 22829319 PMC3440228

[B67] Smyth G. K. (2004). Linear models and empirical bayes methods for assessing differential expression in microarray experiments. *Stat. Appl. Genet. Mol. Biol.* 3 1–25. 10.2202/1544-6115.1027 16646809

[B68] Su L. Diretto G. Purgatto E. Danoun S. Zouine M. Li Z. (2015). Carotenoid accumulation during tomato fruit ripening is modulated by the auxin-ethylene balance. *BMC Plant Biol.* 15:114 10.1186/s12870-015-0495-4 PMC4424491 25953041

[B69] Sun L. Sun Y. Zhang M. Wang L. Ren J. Cui M. (2012). Suppression of 9-cis-epoxycarotenoid dioxygenase, which encodes a key enzyme in abscisic acid biosynthesis, alters fruit texture in transgenic tomato. *Plant Physiol.* 158 283–298. 10.1104/pp.111.186866 22108525 PMC3252109

[B70] Sun L. Zhang M. Ren J. Qi J. Zhang G. Leng P. (2010). Reciprocity between abscisic acid and ethylene at the onset of berry ripening and after harvest. *BMC Plant Biol.* 10:257 10.1186/1471-2229-10-257 PMC3095336 21092180

[B71] Symons G. M. Davies C. Shavrukov Y. Dry I. B. Reid J. B. Thomas M. R. (2006). Grapes on steroids. Brassinosteroids are involved in grape berry ripening. *Plant Physiol.* 140 150–158. 10.1104/pp.105.070706 16361521 PMC1326039

[B72] Takezawa D. Komatsu K. Sakata Y. (2011). ABA in bryophytes: how a universal growth regulator in life became a plant hormone? *J. Plant Res.* 124 437–453. 10.1007/s10265-011-0410-5 21416316

[B73] Tijero V. Teribia N. Muñoz P. Munné-Bosch S. (2016). Implication of abscisic acid on ripening and quality in sweet cherries: differential effects during pre- and post-harvest. *Front. Plant Sci.* 7:602 10.3389/fpls.2016.00602 PMC4855249 27200070

[B74] Vandesompele J. De Preter K. Pattyn F. Poppe B. Van Roy N. De Paepe A. (2002). Accurate normalization of real-time quantitative RT-PCR data by geometric averaging of multiple internal control genes. *Genome Biol.* 3:RESEARCH0034. 10.1186/gb-2002-3-7-research0034 PMC126239 12184808

[B75] Velasco R. Zharkikh A. Troggio M. Cartwright D. A. Cestaro A. Pruss D. (2007). A high quality draft consensus sequence of the genome of a heterozygous grapevine variety. *PLoS ONE* 2:e1326 10.1371/journal.pone.0001326 PMC2147077 18094749

[B76] Wang M. Vannozzi A. Wang G. Liang Y.-H. Tornielli G. B. Zenoni S. (2014). Genome and transcriptome analysis of the grapevine (*Vitis vinifera* L.) WRKY gene family. *Hortic. Res.* 1:14016 10.1038/hortres.2014.16 PMC4596322 26504535

[B77] Wang N. Zheng Y. Xin H. Fang L. Li S. (2013). Comprehensive analysis of NAC domain transcription factor gene family in *Vitis vinifera*. *Plant Cell Rep.* 32 61–75. 10.1007/s00299-012-1340-y 22983198

[B78] Wang P. Xue L. Batelli G. Lee S. Hou Y.-J. Van Oosten M. J. (2013). Quantitative phosphoproteomics identifies SnRK2 protein kinase substrates and reveals the effectors of abscisic acid action. *Proc. Natl. Acad. Sci. U.S.A.* 110 11205–11210. 10.1073/pnas.1308974110 23776212 PMC3703982

[B79] Wanke D. (2011). The ABA-mediated switch between submersed and emersed life-styles in aquatic macrophytes. *J. Plant Res.* 124 467–475. 10.1007/s10265-011-0434-x 21674229

[B80] Wheeler S. Loveys B. Ford C. Davies C. (2009). The relationship between the expression of abscisic acid biosynthesis genes, accumulation of abscisic acid and the promotion of *Vitis vinifera* L. berry ripening by abscisic acid. *Aust. J. Grape Wine Res.* 15 195–204. 10.1111/j.1755-0238.2008.00045.x

[B81] Wong D. C. J. Schlechter R. Vannozzi A. Höll J. Hmmam I. Bogs J. (2016). A systems-oriented analysis of the grapevine R2R3-MYB transcription factor family uncovers new insights into the regulation of stilbene accumulation. *DNA Res.* 23 451–466. 10.1093/dnares/dsw028 PMC5066171 27407139

[B82] Yan T. Yoo D. Berardini T. Z. Mueller L. A. Weems D. C. Weng S. (2005). PatMatch: a program for finding patterns in peptide and nucleotide sequences. *Nucleic Acids Res.* 33 W262–W266. 10.1093/nar/gki368 15980466 PMC1160129

[B83] Yang S.-D. Seo P. J. Yoon H.-K. Park C.-M. (2011). The *Arabidopsis* NAC transcription factor VNI2 integrates abscisic acid signals into leaf senescence via the COR/RD genes. *Plant Cell* 23 2155–2168. 10.1105/tpc.111.084913 21673078 PMC3160032

[B84] Yoshida T. Fujita Y. Sayama H. Kidokoro S. Maruyama K. Mizoi J. (2010). AREB1, AREB2, and ABF3 are master transcription factors that cooperatively regulate ABRE-dependent ABA signaling involved in drought stress tolerance and require ABA for full activation. *Plant J.* 61 672–685. 10.1111/j.1365-313X.2009.04092.x 19947981

[B85] Young P. R. Lashbrooke J. G. Alexandersson E. Jacobson D. Moser C. Velasco R. (2012). The genes and enzymes of the carotenoid metabolic pathway in *Vitis vinifera* L. *BMC Genomics* 13:243 10.1186/1471-2164-13-243 PMC3484060 22702718

[B86] Yu X.-C. Li M.-J. Gao G.-F. Feng H.-Z. Geng X.-Q. Peng C.-C. (2006). Abscisic acid stimulates a calcium-dependent protein kinase in grape berry. *Plant Physiol.* 140 558–579. 10.1104/pp.105.074971 16407437 PMC1361324

[B87] Zhang M. Leng P. Zhang G. Li X. (2009). Cloning and functional analysis of 9-cis-epoxycarotenoid dioxygenase (NCED) genes encoding a key enzyme during abscisic acid biosynthesis from peach and grape fruits. *J. Plant Physiol.* 166 1241–1252. 10.1016/j.jplph.2009.01.013 19307046

