# Supplementary figures and images for: Abscisic Acid Is a Major Regulator of Grape Berry Ripening Onset: New Insights into ABA Signaling Network
Source: Front Plant Sci. 2017 Jun 21;8:1093. doi: 10.3389/fpls.2017.01093 (PMC5479058; doi:10.3389/fpls.2017.01093)

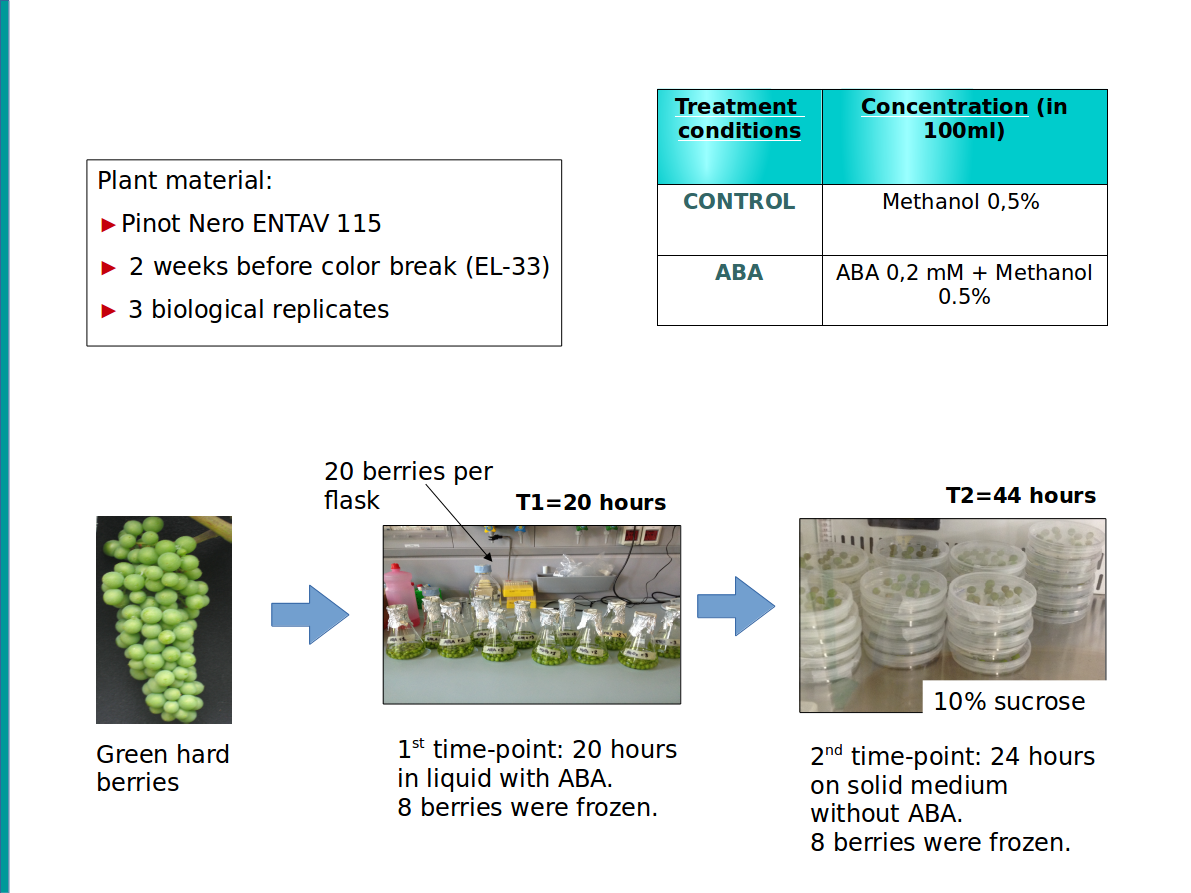

Supplement: FIGURE S1 — Description of the ABA treatment. [file Image_1.TIF]

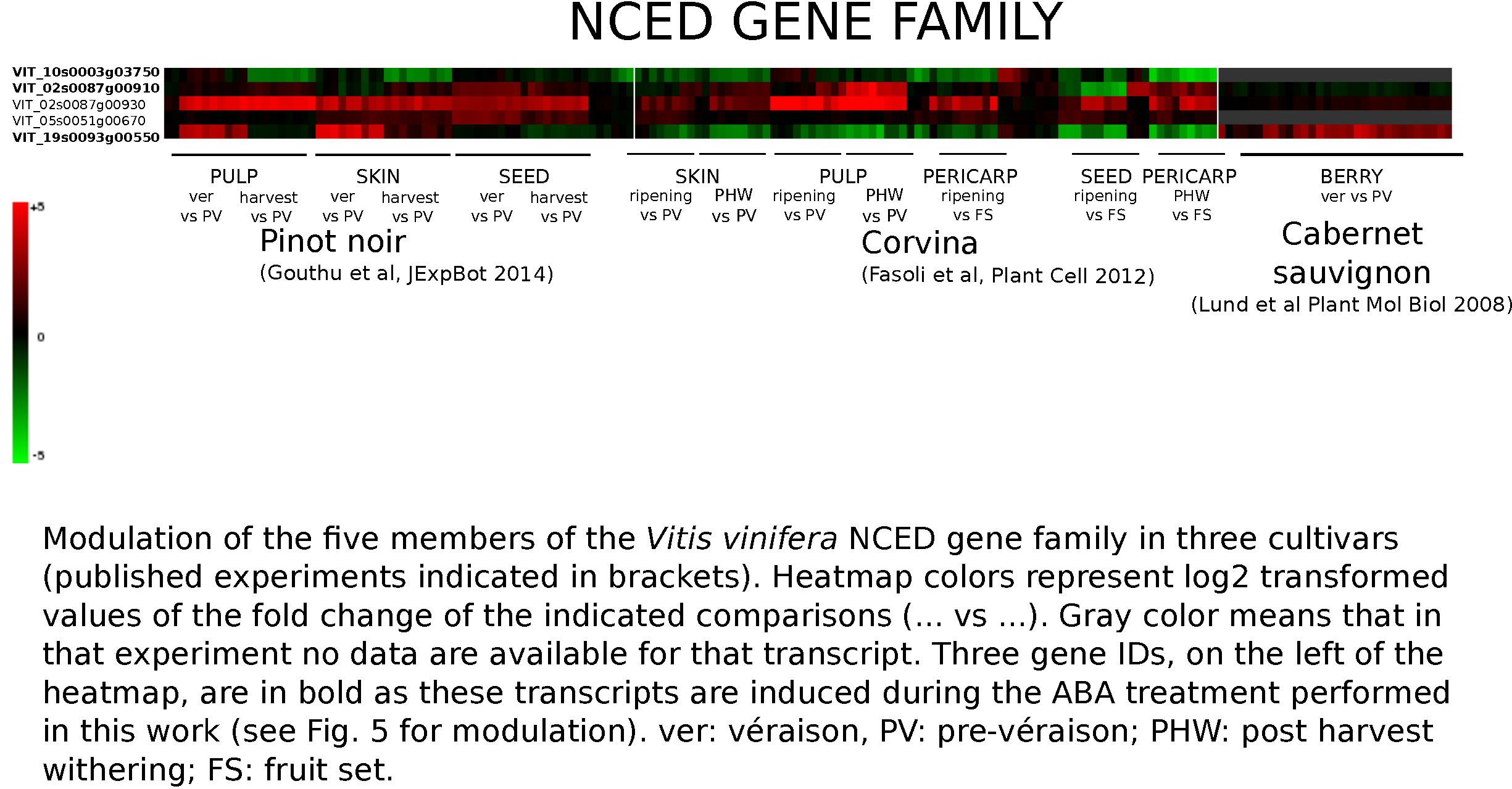

Supplement: FIGURE S2 — Visualization of NCED gene family during berry ripening in three Vitis vinifera cultivars. [file Image_2.TIFF]
